# Promising neuroprotective strategies for traumatic spinal cord injury with a focus on the differential effects among anatomical levels of injury

**DOI:** 10.12688/f1000research.11633.1

**Published:** 2017-10-30

**Authors:** Antigona Ulndreaj, Anna Badner, Michael G Fehlings

**Affiliations:** 1 Institute of Medical Science, University of Toronto, Toronto, Canada; 2 University of Toronto Spine Program, Toronto, Canada; 3 Department of Genetics and Development, Krembil Research Institute, University Health Network, Toronto, Canada

**Keywords:** Traumatic spinal cord injury, neuroprotective treatments, granulocyte colony stimulating fact

## Abstract

Traumatic spinal cord injury (SCI) is a devastating condition of motor, sensory, and autonomic dysfunction. The significant cost associated with the management and lifetime care of patients with SCI also presents a major economic burden. For these reasons, there is a need to develop and translate strategies that can improve outcomes following SCI. Given the challenges in achieving regeneration of the injured spinal cord, neuroprotection has been at the forefront of clinical translation. Yet, despite many preclinical advances, there has been limited translation into the clinic apart from methylprednisolone (which remains controversial), hypertensive therapy to maintain spinal cord perfusion, and early decompressive surgery. While there are several factors related to the limited translational success, including the clinical and mechanistic heterogeneity of human SCI, the misalignment between animal models of SCI and clinical reality continues to be an important factor. Whereas most clinical cases are at the cervical level, only a small fraction of preclinical research is conducted in cervical models of SCI. Therefore, this review highlights the most promising neuroprotective and neural reparative therapeutic strategies undergoing clinical assessment, including riluzole, hypothermia, granulocyte colony-stimulating factor, glibenclamide, minocycline, Cethrin (VX-210), and anti-Nogo-A antibody, and emphasizes their efficacy in relation to the anatomical level of injury. Our hope is that more basic research will be conducted in clinically relevant cervical SCI models in order to expedite the transition of important laboratory discoveries into meaningful treatment options for patients with SCI.

## Introduction

Traumatic spinal cord injury (SCI), which is caused by external mechanical impact, results in impairment of motor, sensory, and autonomic function at and below the level of injury. Mechanical laceration, contusion, and compression result in cell death, which is further propagated by secondary injury mechanisms which include ischemia, sodium- and calcium-mediated cell injury, glutamatergic excitotoxicity, hemorrhage, and inflammation. The secondary injury amplifies the primary damage and promotes cystic degeneration and glial scar formation, thereby preventing functional recovery. Therefore, targeting secondary injury is a promising therapeutic intervention.

Despite several efficacious preclinical studies for SCI, there have been challenges in achieving successful translation into the clinic. While the disconnect between bench and bedside is not limited to SCI, it is important to recognize the underlying factors and identify solutions. Clinical heterogeneity, complexity of the disease, and the limited regenerative capacity of the spinal cord are among the key causes for poor translation and have been broadly discussed in the literature ^1^. Yet significantly less emphasis has been placed on the need to apply clinically relevant models of cervical SCI ^2^. Given that over 50% of human SCI cases occur at the cervical level ^3^ and the majority of preclinical work involves thoracic injuries ( Table 1), translation will require a greater understanding of injury-level subpopulation differences in pathophysiology and therapeutic benefits.

**Table 1.** Experimental evidence for the efficacy of promising neuroprotective therapies.

Neuroprotective/ neural reparative therapy	Injury model, species	Reference
Riluzole	Contusion, T7–T10, rat	69
	Compression, T8, rat	70
	Compression, T6, rat	71
	Contusion, T10, rat	72
	Compression, T11, rat	73
	Contusion, T8, rat	74
	Unilateral contusion, C7, rat	75
	Hemisection, C2, rat	35
	Compression, C7, rat	76
	Unilateral contusion, C7, rat	77
	Compression, C7, rat	78
	Compression, C7, rat	79
	Transection, S2, rat	34
Hypothermia	Contusion, T8, rat	80
	Compression, T8, rat	81
	Compression, T8, rat	82
	Compression, T11, rat	83
	Contusion, T9, rat	84
	Contusion, T10, rat	85
	Unilateral contusion, C7, rat	75
	Contusion, C5, rat	86
Glibenclamide	Contusion, T8, rat	87
	Unilateral contusion, T9, mouse	88
	Unilateral contusion, C7, rat	75
	Unilateral contusion, C7, rat	89
	Contusion, C7, rat	90
	Unilateral contusion, C4, rat	42
	Unilateral contusion, C7, rat	77
	Unilateral contusion, C7, rat	91
Granulocyte colony-stimulating factor	Contusion, T10, rat	92
	Compression, T9, rat	93
	Contusion, T9, rat	94
	Hemisection, T10, mouse	95
	Contusion, T8, rat	96
	Contusion, T9, rat	97
	Contusion, T8, rat	44
	Compression, T8, mouse	98
	Compression, T7, mouse	99
	Transection, T8, mouse	100
	Contusion, T8, rat	101
	Compression, T8, rat	102
Minocycline	Contusion, T7, rat	103
	Contusion, T9, rat	104
	Contusion, T9, mouse	105
	Contusion, T9, rat	106
	Contusion, T9, rat	107
	Hemisection, T13, rat	108
	Contusion, T10, rat	109
	Contusion, T9, rat	110
	Contusion, T10, rat	111
	Contusion, T9, rat	112
	Dorsal transection, C7, rat	113
	Unilateral contusion, C5, rat	114
	Compression, T3, mouse	115
Cethrin (VX-210)	Contusion, T8, mouse	116
	Dorsal hemisection, T7, mouse	117
	Dorsal transection, T3, rat	118
	Contusion, T9, rat	119
Anti-Nogo-A antibody	Hemisection, T10, rat	120
	Dorsolateral hemisection, T8, rat	121
	T-shape transection, T9, rat	122
	Partial hemisection, T8, monkey	123
	T-shape transection, T8, rat	124
	T-shape transection, T8, rat	125
	T-shape transection, T8, rat	126
	Dorsal hemisection, T8, rat	52
	Partial dorsal transection, T6, rat	53
	Partial hemisection, C7, monkey	54
	Hemisection, C7, monkey	55

The table summarizes the model, anatomical level of spinal cord injury, and the species used to evaluate the effectiveness and mechanisms of action of the neuroprotective therapies undergoing clinical trials. Although this list is not exhaustive, it highlights that thoracic models of spinal cord injury are most commonly applied at the preclinical level. All injury models are bilateral if not stated otherwise.

**Figure 1.**
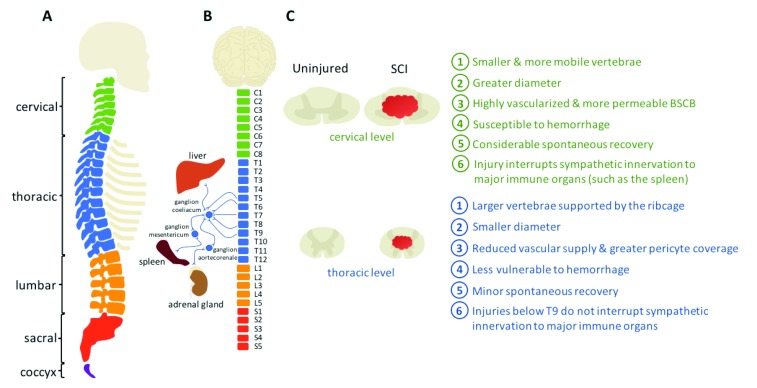
There are several key differences between cervical and thoracic spinal cord injury ( **A**) The cervical vertebrae are smaller and more mobile than their thoracic counterparts, which are further supported by the rib cage. ( **B**) The cervical spinal cord also has a larger diameter, and injuries at the cervical level interrupt the sympathetic innervation to major immune organs. ( **C**) Moreover, the greater vascularity of the cervical cord increases susceptibility to hemorrhage following trauma. Lastly, injuries at the cervical level allow for considerably more spontaneous recovery compared with injuries at the thoracic level ^128^. BSCB, blood spinal cord barrier; SCI, spinal cord injury.

Differences between the cervical and thoracic cord anatomy, physiology, and immune response may affect the outcome of neuroprotective treatments ( Figure 1). Anatomically, the cervical spine has small vertebrae and increased mobility, which make it more susceptible to injury compared to the thoracic region. The cervical spinal cord also has a larger diameter, a greater blood supply, and larger gray and white matter areas ^4^. Relatedly, the cervical gray matter vasculature has less pericyte coverage than the thoracic cord, resulting in a blood spinal cord barrier predisposed to increased permeability ^5, 6^. Also, some of the most frequent conditions of the spine, such as central cord syndrome ^7^ and degenerative cervical myelopathy ^8^, affect primarily the cervical region. Of note, while the cervical spinal cord is especially vulnerable to injury and hemorrhage, it may also be more accessible to systemically administered therapeutics. Adding to this complexity, there is emerging evidence demonstrating level-dependent variations in the immune response ^9, 10^. For example, interestingly, higher-level injuries may be less prone to chronic autoimmunity ^11, 12^. Therefore, as SCI pathophysiology may differ between anatomical levels of injury, there is growing awareness that treatments should be tailored to the patient’s injury. Here, we review the most promising neuroprotective approaches, emphasizing their effect differences based on the level of injury ( Table 2).

**Table 2.** Neuroprotective strategies currently in clinical trials.

Neuroprotective/ neural-reparative drug	ClinicalTrials.gov identifier	Status	Enrollment (number of patients, level of injury		Results	Mechanism of action	Reference
			Thoracic	Cervical			
Riluzole	NCT00876889	Completed	8 (T1–T11)	28 (C4–C8)	Motor score improvement in patients with cervical SCI, particularly with incomplete injuries. No significant effect in patients with thoracic SCI.	Limit excitotoxicity	36
	NCT01597518	Recruiting	0	Est. enrollment 351 (C4–C8)			37
Therapeutic hypothermia	N/A	Completed	0	14 (C4–C7)	Trend toward improvement of motor scores compared with historical controls in the same institution.	Reduce excitotoxicity, inflammation, and vasogenic edema	40
	NCT02991690	Recruiting	0	Est. 120 (C1–C8)			ClinicalTrials.gov
Glibenclamide	NCT02524379	Recruiting	0	Est. 10 (C2–C8)		Limit hemorrhage	ClinicalTrials.gov
Granulocyte colony- stimulating factor	N/A	Completed	0	28 (C2–C6)	Motor score improvement at 3 months of treatment compared with MPSS historical controls.	Promote neurogenesis and angiogenesis, and reduce inflammation	46
	N/A	Completed	0	17 (C2–C6)	Motor score improvement from 1 week to 1 year after SCI compared with placebo in an open non-randomized trial.		47
Minocycline	NCT00559494	Completed	17 (T1–T12)	25 (C1–C8)	Motor score improvement in patients with cervical SCI, particularly for motor incomplete. No benefit for patients with thoracic SCI.	Reduce inflammation	49
	NCT01828203	Recruiting	0	Est. 248 (C0–C8)			ClinicalTrials.gov
Cethrin (VX-210)	NCT00500812	Completed	32 (T2–T12)	16 (C4–T1)	Significant motor score improvement in patients with cervical injury. No effect in patients with thoracic SCI.	Inhibit axonal dieback and reduce inflammation	50
	NCT02669849	Recruiting	0	Est. 150 (C5–C6)			ClinicalTrials.gov
Anti-Nogo-A antibody	NCT00406016	Completed	52 patients with injury between C5 and T12; no data available about injury level classification		No adverse effects.	Promote neurite sprouting	127
							ClinicalTrials.gov

The table lists the discussed neuroprotective strategies for spinal cord injury (SCI) undergoing clinical evaluation. The status of trials and enrollment information, including level of injury and results, are summarized. This demonstrates that clinical trials are predominately focused on cervical SCI. Est, estimated; N/A, not applicable; MPSS, methylprednisolone sodium succinate.

## Neuroprotective strategies in current care

### Early surgical decompression

The popularized phrase coined by the senior author, “Time is spine”, highlights the preclinical ^13, 14^ and clinical ^15, 16^ success of early surgical decompression, which aims to realign the spinal column and relieve bony or ligamentous spinal cord compression. Decompression of intradural pressure, by durotomy alone or durotomy combined with duraplasty, has also been evaluated in experimental SCI ^17, 18^. Yet mixed results warrant further research on the efficacy and standardization of these practices. In contrast, early extradural surgical decompression has been shown to reduce tissue damage and improve outcomes following SCI. Even with some concern regarding perioperative hemodynamic changes affecting cord perfusion, most spine surgeons have been, and continue to be, in favor of decompressing the acutely injured spinal cord ^19, 20^. As a result, early decompression remains recommended in clinical management guidelines by the American Association of Neurological Surgeons (AANS) and the Congress of Neurological Surgeons ^21^. Similarly, the recent AOSpine guideline also recommends decompression within 24 hours of SCI ^22^. While there may be differential efficacy between injury-level subpopulations ^23^, current evidence is limited by substantial clinical heterogeneity, loss to follow-up, unclear adjustment for baseline factors, and a lack of statistical power. For these reasons, more work is needed to develop customizable treatment regimens and prioritized surgical access to the most benefitting patient subtypes.

### Support of mean arterial pressure

Hypotension, hypoxemia, pulmonary dysfunction, and cardiovascular instability are common within the first 7 to 10 days of SCI ^24^. Hemodynamic instability not only limits the opportunity for early surgical intervention but also increases spinal cord ischemia and therefore secondary damage. For this reason, the current AANS and Congress of Neurological Surgeons guideline recommends continuous hemodynamic monitoring, interventions correcting hypotension (such as by vasopressor administration), and maintenance of mean arterial blood pressure (MAP) between 85 and 90 mmHg for the first 7 days following cervical injury ^25^. While these recommendations are largely based on a small group of uncontrolled and underpowered studies ^26^, a recent retrospective assessment largely confirmed the published guidelines as well as the neuroprotective potential of vasopressor administration ^27^. As these results need further prospective validation, it will be important to stratify patient populations and identify potential treatment effect differences based on the anatomical level of injury. It is also important to note that MAP support principally aims to maintain appropriate spinal cord perfusion pressure (SCPP), determined by the difference between MAP and intraspinal pressure (ISP). However, as ISP may increase independently of MAP, maintenance of a low ISP or a high SCPP (or both) is gaining increasing attention as an important practice in the acute clinical management of SCI ^28, 29^. While initial studies have shown encouraging results about the predictive value of low ISP or high SCPP in neurological recovery, larger multicenter studies are needed to validate these preliminary data ^29^.

### Methylprednisolone sodium succinate

Methylprednisolone sodium succinate (MPSS) is a synthetic corticosteroid with potent anti-inflammatory effects and neuroprotective potential in acute traumatic SCI. Concerns about increased risk for infections following MPSS treatment have kept the drug at the forefront of continuous controversy. While it remains the only treatment option for acute SCI, debate regarding optimal dose, time of administration, efficacy, and adverse effects has dominated the field for decades and has dichotomized clinicians around the world. For this reason, there have been three National Acute Spinal Cord Injury Studies (NASCIS) to evaluate the clinical safety and efficacy of varying MPSS dose and timing. Moreover, NASCIS results have been retrospectively analyzed on numerous occasions to derive meaningful conclusions. One of the most recent publications on the topic concluded that MPSS does not increase the risk of infections and confers significant short-term effects when given within the first 8 hours of injury ^30^. Importantly, patients with cervical SCI and reduced baseline injury severity seem to benefit most from this treatment ^31^. Given the particularly debilitating nature of cervical injuries, these improvements have tremendous impact on patients’ quality of life. Thus, the most recent AOSpine guideline currently recommends a 24-hour treatment of intravenous MPSS when initiated within the first 8 hours of SCI, independently of injury level ^22^.

## Promising neuroprotective and neural reparative therapies in clinical trials

### Riluzole

Secondary injury involves ionic dysregulation and excitotoxicity. As cell membranes become highly permeable to sodium ions, there is increased calcium influx. Subsequently, high sodium and calcium ion concentrations in neurons trigger the secretion of glutamate from nerve terminals. Increased synaptic glutamate leads to prolonged excitability in the postsynaptic neurons, driving eventual neuronal edema and death.

Riluzole is a benzothiazole (molecular weight of 234.2 Da) which inhibits voltage-gated sodium channels and glutamate release, thereby mitigating excitotoxicity. Riluzole has been reported to slow the progression of amyotrophic lateral sclerosis (ALS)—a progressive motor neuron disease—and currently is the only US Food and Drug Administration (FDA)-approved drug for ALS. In addition, riluzole has been shown to have neuroprotective potential in animal models of Parkinson’s disease ^32^ and Huntington’s disease ^33^ and currently is being used in clinical studies of mild Alzheimer’s disease (ClinicalTrials.gov identifier NCT01703117). Importantly, riluzole was also shown to suppress spasticity ^34^, a frequent co-morbidity in patients with SCI, and to promote neural preservation in rats with high cervical spinal hemisection injury ^35^.

Capitalizing on the preclinical success of riluzole, a phase I/IIA clinical trial was launched in April 2010 to assess the safety and pharmacokinetics of riluzole in patients with acute traumatic SCI (ClinicalTrials.gov identifier NCT00876889). In this trial, 36 patients with SCI (28 cervical and eight thoracic SCI) received riluzole (50 mg) orally every 12 hours for 28 doses. A control group consisting of 36 patients with SCI—matched for neurological impairment, gender, and age—received the standard of care but no riluzole. Patients who received riluzole showed statistically significant improvement compared with the control group ( *P* = 0.021). In particular, patients with incomplete cervical injury—American Spinal Injury Association (ASIA) Impairment Scale B—showed the highest improvement in the International Standards for Neurologic Classification of Spinal Cord Injury (ISNCSCI) motor score ( *P* = 0.037). In addition to being efficacious, riluzole was shown to be safe for this patient cohort ^36^. Interestingly, there was no difference within the thoracic injury group, as patient numbers were small, patients had more severe injuries, and the ISNCSCI motor scoring is less sensitive to thoracic recovery ^37^.

Based on these results, a phase IIB/III was launched in 2013 to evaluate the efficacy and safety of riluzole in patients with cervical traumatic SCI, entitled “Riluzole in Acute SCI Study” (RISCIS) (estimated enrollment: 351 patients, ClinicalTrials.gov identifier NCT01597518). In this multicenter, randomized, placebo-controlled, double-blinded trial, riluzole (100 mg, twice daily) is administered orally to patients within 24 hours from injury, followed by two 50 mg daily doses for 14 days after SCI. A capsule identical in shape and size to riluzole is administered to patients in the control group. The primary outcome of the study is improvement in ISNCSCI motor scores at 180 days after injury. The study is estimated to be completed by 2021 ^37^.

### Therapeutic hypothermia

In response to trauma, increased metabolic rate can lead to excitotoxicity and cell death. Local or systemic cooling following insult has been shown to reduce the metabolic demand, thereby limiting cell death. Moreover, therapeutic hypothermia has been shown to reduce inflammatory cell infiltration, myeloperoxidase activity, and vasogenic edema and stabilize the blood-brain barrier ^38^. Despite these benefits, systemic hypothermia may have some serious side effects, including bradycardia, respiratory infections, and deep vein thrombosis. While local cooling of the spinal cord circumvents many of these concomitant issues, randomized controlled trials are still needed to prove the efficacy of local hypothermia in neurological recovery after SCI. However, in one study, acute (within 8 hours of injury) local hypothermia was shown to improve recovery among cervical and thoracic populations (n = 12 out of 14 cervical SCI, n = 4 out of 6 thoracic SCI) when compared with historical controls ^39^. Similarly, a pilot study of systemic hypothermia in patients with cervical complete SCI (n = 14) demonstrated fewer adverse effects and a trend toward improved recovery compared with age- and injury level-matched historical controls when systemic hypothermia was induced within 9 hours of trauma ^40^. Furthermore, a follow-up randomized controlled trial assessing the efficacy of intravascularly delivered systemic hypothermia in acute cervical SCI commenced in May 2017 (estimated enrollment: 120 patients, ClinicalTrials.gov identifier NCT02991690). Although previous multicenter randomized clinical trials found hypothermia to be ineffective in adults with traumatic brain injury ^41^, expectations for SCI remain hopeful.

### Glibenclamide (Glyburide, DiaBeta)

Capillary fragmentation following SCI contributes to hemorrhage. This process is initiated in the capillary-rich gray matter of the injury epicenter and expands rostro-caudally, leading to progressive tissue necrosis, cavitation, and neurological dysfunction. In a rat model of unilateral cervical SCI, Simard *et al*. found that sulfonylurea receptor 1 (SUR1)-regulated Ca ^2+^-activated [ATP] _i_-sensitive non-specific cation (NC _Ca-ATP_) channels of the capillary endothelium in the spinal cord are key to capillary fragmentation following SCI ^42^. By blocking NC _Ca-ATP_ channels with the FDA-approved anti-diabetic drug glibenclamide (Glyburide), Simard *et al*. observed decreased lesion volumes and significant white matter preservation coupled with improved neurobehavioral outcomes ^42^. Recently, a phase I/II clinical trial was initiated to assess the safety and neuroprotective effectiveness of Glyburide (DiaBeta) in patients with acute traumatic cervical SCI (estimated enrollment: 10 patients, ClinicalTrials.gov identifier NCT02524379), with an estimated completion date in early 2020.

### Granulocyte colony-stimulating factor

Initially overlooked for its potential in the central nervous system (CNS), granulocyte colony-stimulating factor (G-CSF) has shown positive preclinical results for SCI ( Table 1). In response to ischemia and CNS injury, G-CSF and its receptor (CD114; G-CSFR) are upregulated in neurons and endogenous stem cells, initiating a compensatory neuroprotective mechanism. By binding to its cognate receptor, G-CSF counteracts programmed cell death in mature neurons, induces neurogenesis, and promotes neuronal differentiation of adult neural stem cells ^43^. Moreover, angiogenesis ^44^ and reduced inflammation ^45^ have been attributed to the protective actions of G-CSF. Kamiya *et al*. administered G-CSF for 5 consecutive days after cervical SCI and assessed ASIA motor scores 3 months later ^46^. The improvements were significant compared with historical controls of patients with cervical SCI receiving high-dose MPSS ^46^. In a study by Inada *et al*., patients with cervical SCI who received G-CSF demonstrated improved recovery compared with a non-treated group ^47^. However, the treatment was administered in an open-label and non-randomized fashion ^47^. Interestingly, in a study by Saberi *et al*., in which G-CSF was administered in patients with chronic SCI, significant motor and sensory recovery was demonstrated, particularly in patients with incomplete cervical SCI ^48^. Despite these promising effects, a true double-blinded randomized control clinical trial for G-CSF has yet to be developed.

### Minocycline

Inflammatory cytokines produced by resident microglia and astrocytes following trauma attract peripheral immune cells to the spinal cord. Neutrophils and monocytes are the first blood-derived cells to enter the injured parenchyma. While these cells are crucial in cleaning up the cellular debris, they produce inflammatory cytokines, such as tumor necrosis factor-alpha and interferon-gamma, as well as toxic by-products that exacerbate damage.

Minocycline is a tetracycline antibiotic with neuroprotective and anti-inflammatory properties. A single-center, placebo-controlled, double-blinded phase I/II clinical trial was initiated in 2004 to evaluate the efficacy and safety of intravenous minocycline within 12 hours of injury for 7 days. The study, which was completed in 2010 (27 patients received minocycline and 25 received placebo), showed a trend toward improved motor scores in incomplete cervical SCI cases in the absence of any serious adverse effects ( *P* = 0.05) but no improvement in thoracic SCI ^49^ (ClinicalTrials.gov identifier NCT00559494). Based on these results, a phase III clinical trial, titled “Minocycline in Acute Spinal Cord Injury (MASC)”, was initiated in 2013 and is expected to finish by 2018 (estimated enrollment: 248 patients, ClinicalTrials.gov identifier NCT01828203). Interestingly, a clinical trial evaluating the efficacy of minocycline in reducing neuropathic pain has been successfully completed, but the results have yet to be published (ClinicalTrials.gov identifier NCT01869907). Given that neuropathic pain is a common and debilitating co-morbidity in patients with SCI, the study results will be of significant interest to the field.

### Cethrin (VX-210)

The injured spinal cord niche contains growth-inhibitory molecules, such as myelin debris and chondroitin sulfate proteoglycans, that lead to neuron growth cone collapse, thereby inhibiting regeneration. These molecules bind to respective receptors on regenerating neurons, where they initiate a phosphorylation cascade. At the converging point of this cascade are Rho GTPases, a family of intracellular enzymes that regulate cytoskeletal mechanisms and cellular mobility. Cethrin (VX-210) is a recombinant deactivator of RhoA (a member of the Rho family) with dura and cell membrane penetrance. An open-label uncontrolled phase I/IIa clinical trial showed significant neurological improvement in patients with SCI who received Cethrin (48 patients, ClinicalTrials.gov identifier NCT00500812). Benefits were particularly enhanced in patients with cervical SCI compared with their thoracic counterparts ^50^, a finding that incentivized the initiation of larger controlled double-blinded clinical trials for patients with cervical SCI. This phase IIb/III clinical trial will evaluate the safety and efficacy of two doses of VX-210 (formerly known as Cethrin) compared with placebo (a fibrin sealant) when applied extradurally at the site of injury, acutely after cervical SCI (estimated enrollment: 150 patients, ClinicalTrials.gov identifier NCT02669849).

### Anti-Nogo-A antibody (ATI-355)

Anti-Nogo-A antibody is a monoclonal antibody against Nogo-A, a protein inhibitor of neurite growth found on adult CNS myelin. Widely assessed at the thoracic level in rodents ^51– 53^, in addition to several studies in primate models of cervical SCI ^54, 55^, anti-Nogo-A antibody has been shown to promote axonal sprouting and improve functional recovery following injury. A non-randomized, open-label phase I clinical trial of humanized anti-Nogo-A antibody (ATI-355; Novartis Pharmaceuticals) was initiated to assess the feasibility, tolerability, and safety of either repeated intrathecal bolus injections of AT1-355 or continuous intrathecal delivery in acute SCI (4–14 days after injury). In total, 52 cervical and thoracic patients with traumas between C5 and T12 level were recruited in the study, and results are pending dissemination (ClinicalTrials.gov identifier NCT00406016). A phase IIb trial, led by Armin Curt, is expected to begin in Europe shortly.

## Emerging neuroprotective approaches

### Intravenous immunoglobulin G

Intravenous immunoglobulin G (IVIG) consists of serum immunoglobulin G (IgG) pooled from thousands of healthy donors. Independent laboratory studies in cervical and thoracic models of SCI have shown that IVIG improves recovery by targeting the detrimental inflammatory response in the spinal cord after trauma ^56– 58^. While the efficacy of IVIG for SCI has not been assessed in clinical trials, the exciting preclinical results coupled with IVIG’s long-term clinical use for the treatment of autoimmune and immunodeficiency conditions make it a promising candidate for SCI clinical trials.

### Cell therapies

With cell transplantation as an attractive treatment approach for SCI, a diverse range of cells has been evaluated in preclinical studies, resulting in a plethora of potential mechanisms. In short, transplanted cells have been used for immune modulation, trophic support, scaffolding, re-myelination, and cell replacement ^59^. Yet, predominantly applied in the subacute and chronic phases of injury, only a few cell transplantation strategies are thought to have neuroprotective potential for the acutely injured spinal cord.

***Mesenchymal stem/stromal cells.*** Mesenchymal stem/stromal cells (MSCs) are multipotent mesodermal progenitors defined by their *in vitro* adhesion to plastic and their cell surface antigen profile ^60^. Readily accessible from various adult tissues such as bone marrow, cartilage, and fat, MSCs are among the most commonly studied cells in regenerative medicine. This popularity has led to significant heterogeneity in MSC isolation, cultivation, and purification procedures, further resulting in mixed therapeutic efficacy among preclinical studies and the increasing number of clinical studies ^61^. In SCI, MSCs have been reported to dampen inflammation, modulate the immune response, and secrete neuroprotective factors ^59^. A 2013 systematic meta-analysis of preclinical studies, involving intrathecal, intraparenchymal, and intravenous infusion of MSCs in various models of cervical and thoracic SCI, determined that the cells, overall, result in improved functional recovery after injury ^62^. While this is encouraging, a lot remains to be understood about the identity and function of MSCs. Furthermore, additional mechanistic studies are needed to effectively tailor their therapeutic application for SCI and identify differences in efficacy between anatomical levels of injury.

## Final thoughts

Spinal cord level-dependent differences in vertebral structure, anatomy, and peripheral immune organ innervation may affect SCI pathophysiology. Though largely overlooked in preclinical studies, post hoc subgroup analysis from seminal large-scale clinical trials has been indicative of varying treatment efficacy between different anatomical levels of SCI ^50, 63^. As a result, the stratification of patients into injury-level subpopulations is being increasingly adopted in trial design, and the aforementioned RISCIS trial is a leading example ^37^.

Notwithstanding its strengths, this approach has considerable challenges. Firstly, given the clinical heterogeneity of SCI pathophysiology, even within the same level of injury, the recruitment of adequate patient numbers to reach statistical power may prove substantially difficult, especially for acute and infrequent injuries. Although multicenter trials may circumvent this issue, they require tremendous coordination, collaboration, and resources. However, a recently published assessment of patient recruitment for acute SCI trials determined that such multicenter Canadian trials are feasible with careful *a priori* planning and registry support ^64^. Secondly, stratified analysis is susceptible to confounding effects. For instance, treatment efficacy may be directly affected by patient age or injury causation rather than the anatomical level of injury ^3^. In addition, stratification according to injury level alone is unlikely to account for the significant variability in injury severity, presentation, and patient characteristics. Finally, a greater understanding of the impact of trauma-related mechanisms on the impact and outcomes of SCI is also required.

Therefore, it is increasingly recognized that optimal patient recovery will stem from a combinatorial treatment regimen of integrated pharmacological and rehabilitation-based strategies that will be personalized to their SCI signature (age, medical history, level, completeness, and mechanism of injury). For this reason, translational laboratory studies need to compare neuroprotective efficacy, as well as combinatorial approaches, between different anatomical levels of injury and severity. Moreover, advances in imaging and biochemical biomarkers are needed to help tailor trials within a heterogeneous SCI patient population, narrowing the inclusion window and increasing study power. These approaches can be further applied to better assess treatment efficacy, specifically beyond basic neurological recovery. Apart from outcomes, treatment protocols should ensure sufficient drug delivery to target sites, especially for systemically administered neuroprotective agents with CNS-specific effects ^65^. Moreover, coordinated efforts should be made by leading SCI care units to standardize early medical management ^66, 67^ and monitoring practices ^68^ in order to maximize the efficacy of randomized controlled trials. Lastly, with the increasing incidence of traumatic SCI in the elderly ^3^, it is important that more emphasis be placed on optimizing such practices to address the specific needs of this growing demographic.

In conclusion, neuroprotection has the potential to improve recovery of motor, sensory, and autonomic function following SCI. Significant strides in our understanding of SCI pathophysiology, patient presentation, and biomarkers will further align preclinical research with clinical reality, yielding translatable solutions that can benefit patients.
